# Mortality patterns in chronic granulomatous invasive fungal rhinosinusitis: insights from two fatal cases

**DOI:** 10.1093/jscr/rjae770

**Published:** 2024-12-12

**Authors:** Hussain J Aljubran, Omar A Bamalan, Maria R Alabdulaal, Fadhel Almolani, Mohammad S Alahmari, Abdulrahman Alkhatib, Ali Almomen

**Affiliations:** College of Medicine, Imam Abdulrahman Bin Faisal University, Dammam 43221, Saudi Arabia; Department of Surgery, Security Forces Hospital, Dammam 43221, Saudi Arabia; College of Medicine, Arabian Gulf University, Manama 973, Bahrain; Department of Radiology, King Fahad Specialist Hospital, Dammam 32253, Saudi Arabia; Department of Otolaryngology Head and Neck Surgery, King Fahad Specialist Hospital, Dammam 32253, Saudi Arabia; Department of Otolaryngology Head and Neck Surgery, King Fahad Specialist Hospital, Dammam 32253, Saudi Arabia; Department of Otolaryngology Head and Neck Surgery, King Fahad Specialist Hospital, Dammam 32253, Saudi Arabia

**Keywords:** invasive fungal rhinosinusitis, granulomatous, mortality, paranasal sinus diseases

## Abstract

Chronic granulomatous invasive fungal rhinosinusitis (CGIFRS) is a type of invasive fungal rhinosinusitis that is characterized by the presence of pathologic findings of non-caseating granulomas in the paranasal sinuses. This article describes two cases of CGIFRS with fatal outcomes. The first case was for a 36-year-old man who presented with headache, dizziness, and vomiting for 1 month. This patient had received the traditional treatment, although the patient’s condition suddenly deteriorated after 1 week of surgery and died due to the disease’s complications. Similarly, the second case was for a 31-year-old man who presented with bilateral nasal obstruction and left eye proptosis for 1 year. This patient had a recurrence of CGIFRS after the first presentation, which was complicated by a cerebral abscess after 2 months of surgery. This study, therefore, underscores the severity of CGIFRS as a potentially fatal disease.

## Introduction

For a long time, fungal rhinosinusitis has been documented as primarily affecting immunocompromised individuals. However, with the advent of nasal endoscopy, the disease has also been observed in numerous patients with healthy immune function [1]. A new classification system adapted by Rupa *et al.* divides fungal rhinosinusitis into non-invasive, invasive, and mixed fungal rhinosinusitis based on the capacity of the fungal hyphae to infiltrate tissues via the epithelial layer [2]. The non-invasive fungal rhinosinusitis is further categorized as saprophytic fungal infestation, fungal ball, and allergic fungal rhinosinusitis. At the same time, the invasive form is subdivided into acute invasive fungal rhinosinusitis, chronic invasive fungal rhinosinusitis, and chronic granulomatous invasive fungal rhinosinusitis (CGIFRS) [3].

CGIFRS is characterized by a prolonged clinical course of ˃3 months with slow disease progression across mucosa and bone or other contiguous structures with the formation of non-caseating granulomas in the host soft tissues [4]. It is a rare disease that is most commonly caused by *Aspergillus flavus*, with most cases occurring in tropical regions (e.g. India, Sudan, Pakistan) [5, 6]. CGIFRS initially tends to be asymptomatic until it presents at an advanced stage, which means that mortality is a matter of critical concern, particularly because of the increased likelihood of intracranial invasion [7]. In view of the rarity of this disease and its potentially severe outcomes, we present two cases of CGIFRS with fatal outcomes and provide a summary of the literature.

## Case series

### Case 1

**Figure 1:**
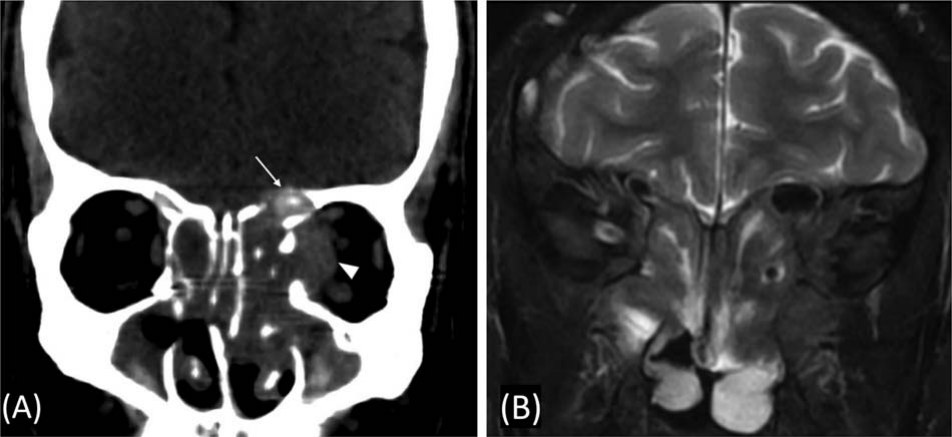
(A) Paranasal sinuses CT scan without contrast showing opacification of the sinuses with hyperdense contents. It results dehiscence of the left lamina papyracea and extension in the medial extraconal space (arrowhead). The ethmoid air cells roof show dehiscence of the roof with intracranial extension (arrow). (B) Coronal T2 MRI through the posterior aspect of left orbit shows the intra-orbital extraconal extension as well as the hypointense left ethmoidal disease with extradural intracranial extension.

**Figure 2:**
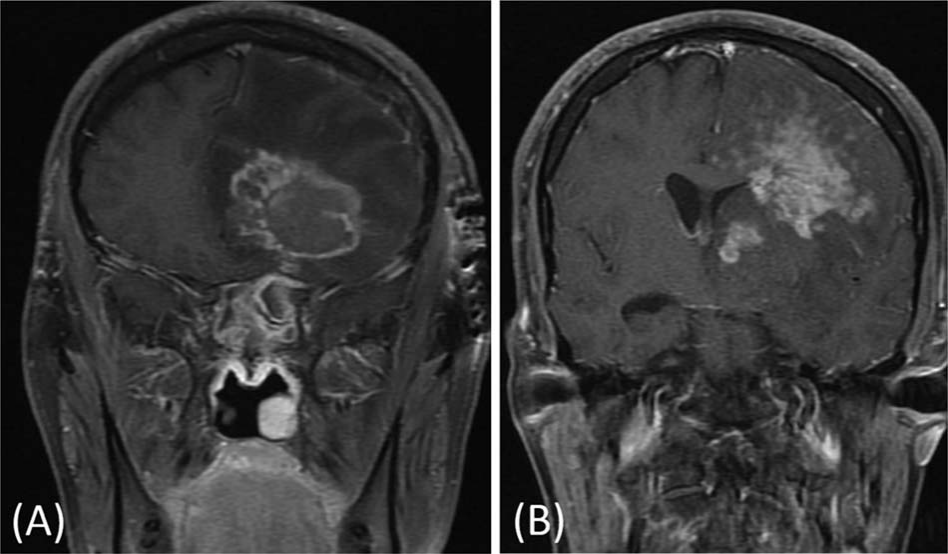
(A and B) Brain MRI with contrast showing signs of intracranial abscess in the left side with mass effect and midline shift to the right side.

A previously healthy 36-year-old man presented to the emergency department complaining of persistent frontal headache, dizziness, and vomiting of 1 month duration, with no history of orbital complaints (e.g. visual deficits or swelling) or neurological deficits (e.g. limb weakness or seizure-like movements). On examination, the nasal endoscopy showed dry crusts filling the right nasal area. Therefore, due to the subtle symptoms’ origin, the patient was admitted, and a computed tomography (CT) scan of the head and a magnetic resonance imaging (MRI) of the brain were scheduled. The head CT scan revealed complete opacification of the maxillary, ethmoid, frontal, and sphenoid sinuses with bone destruction affecting the left lamina papyracea and roof of the ethmoid air cells (Fig. 1A). The brain MRI demonstrated similar findings with intra-orbital extension resulting in left global proptosis, which triggered concerns regarding fungal rhinosinusitis (Fig. 1B). Accordingly, the patient underwent functional endoscopic sinus surgery (FESS) with a right frontal craniotomy and partial removal of the infected brain tissue on the fourth day of his presentation. Specimens from both surgical sites were sent for pathology and cultures during the surgery. The histopathological results confirmed the diagnosis of CGIFRS, and *A. flavus* was found in the tissue culture. Postoperatively, the patient was stable, and intravenous antifungal treatment (amphotericin B and voriconazole) and high-dose steroid administration (dexamethasone) were started promptly following confirmation of CGIFRS via pathology. One week after the surgery, the patient’s condition suddenly deteriorated: he had a right fixed dilated pupil and spikes of fever. An urgent brain MRI with contrast was performed, which showed an intracranial abscess, and a significant midline shift to the right side (Fig. 2A and B). The patient, therefore, had a right decompression craniotomy and remained intubated and ventilated in the intensive care unit to receive the maximum medical therapy. However, despite this intensive treatment, the patient’s condition continued to worsen, and he ultimately died after 2 weeks.

### Case 2

**Figure 3:**
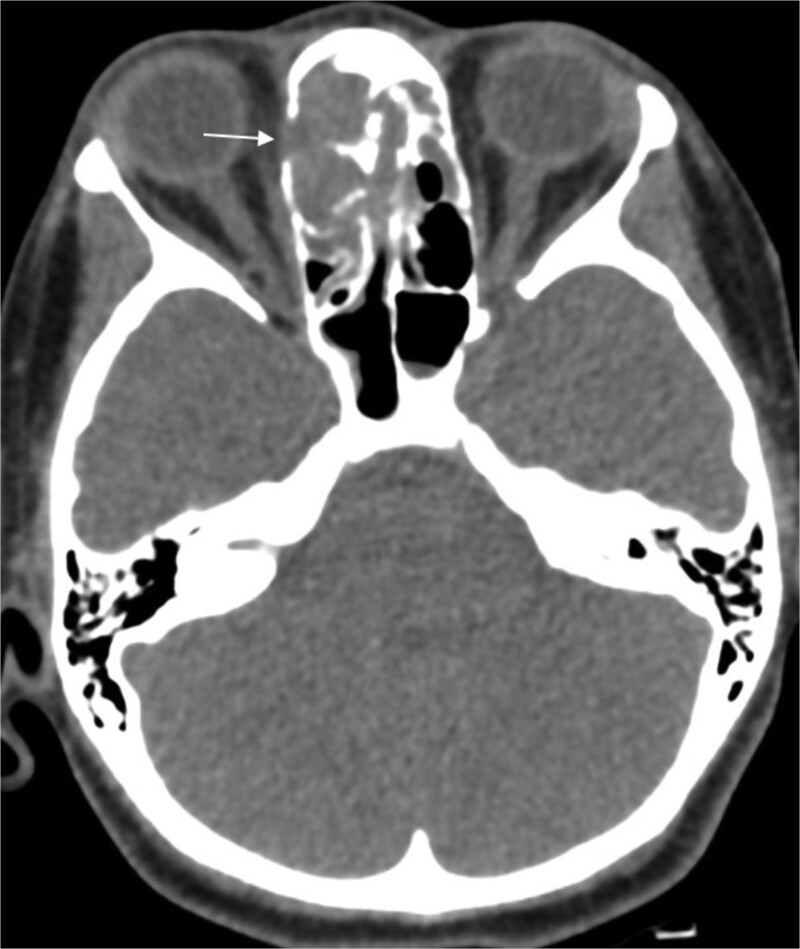
Paranasal CT scan without contrast a mass in the left ethmoid cavity with erosion of the lamina papyracea (arrow).

A 31-year-old man, who is a known case of Gilbert syndrome, presented to the rhinology clinic with a 1-year history of progressive bilateral nasal obstruction and left eye proptosis. The patient had previously undergone FESS twice outside the hospital for allergic fungal rhinosinusitis, with the most recent procedure having been performed two years before his presentation at the clinic. On examination, the nasal endoscopy showed extensive nasal polyposis, while the neurological examinations were normal. The CT scan and MRI revealed a mass in the left ethmoid cavity with an erosion of the lamina papyracea, extending into the extraconal fat and obstructing the frontal recess with mucosal thickening (Fig. 3). The patient was therefore suspected of having recurrent fungal rhinosinusitis and underwent FESS for debulking and to obtain a biopsy. Postoperatively, the histopathological results confirmed the diagnosis of CGIFRS, and *A. flavus* was found in the tissue culture. The patient was then started on oral voriconazole for 3 months.

**Figure 4:**
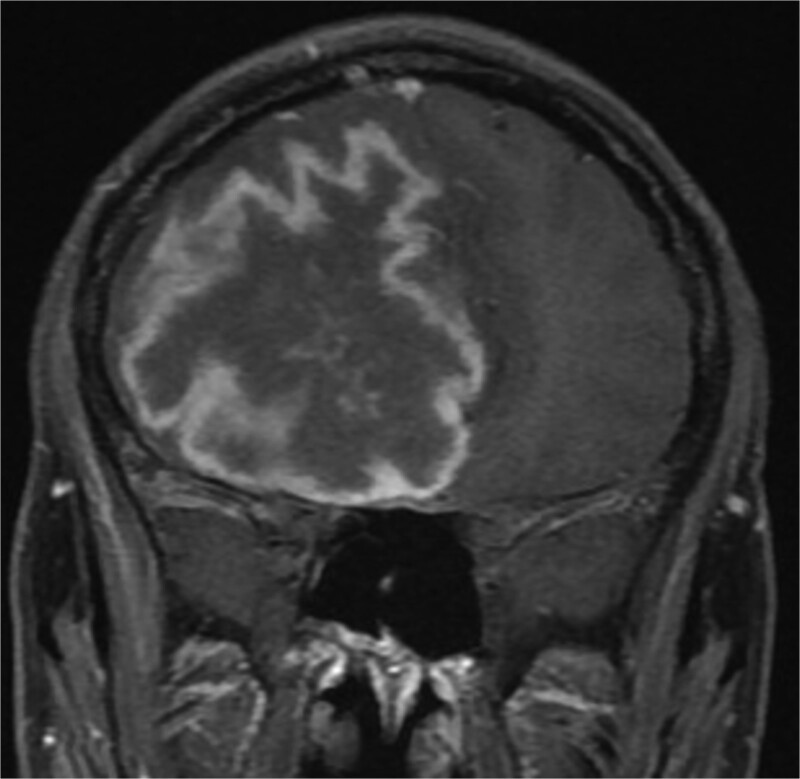
Brain MRI showing right-sided cerebral abscess with midline shift to the left side.

One year later, the patient presented to the emergency department with dizziness, vomiting, and headaches that had started 20 days prior. The CT scan and MRI showed orbital and intracranial invasion. Subsequently, the patient underwent FESS revision with an open craniotomy. The histopathological results revealed findings similar to the first presentation, so the patient was started on intravenous amphotericin B, voriconazole, and dexamethasone. The patient was stable postoperatively until 2 months when he began to deteriorate, and a brain MRI revealed a cerebral abscess (Fig. 4). The patient underwent an urgent open craniotomy with drainage and was shifted to the intensive care unit. However, his condition continued to worsen, and he passed away after 1 month.

## Discussion

Invasive fungal rhinosinusitis is defined based on the presence of fungal hyphae within the host soft tissue on histopathology, as it can be either acute (˂3 months) or chronic (˃3 months). It is further subdivided into non-granulomatous and granulomatous types [8]. Most are immunocompromised individuals; however, it is noteworthy that the granulomatous type is more dominant in immunocompetent people [9]. Although CGIFRS is rare, it must be treated seriously, as it is an aggressive disease with fatal outcomes, and we ought to explore the factors that may contribute to the risk of mortality in this disease.

The risk factors for CGIFRS include living in a warm and dry region, as these areas provide an ideal environment for fungal growth and spore proliferation. Moreover, being involved in agricultural activities raises the risk of significant exposure to fungal spores [10]. The most common causative organism related to CGIFRS is *A. flavus*, although ˃23 organisms have been linked to fungal rhinosinusitis in general [11, 12]. In a recent systematic review involving 255 cases of CGIFRS, *A. flavus* was found to be the most frequent causative organism in 64% of the cases [2]. The predominant patient age group for CGIFRS is between 35 and 49 years, with the majority (67.7%) of cases occurring in men [11, 13]. A high level of suspicion is needed to diagnose patients with CGIFRS as symptoms specifically associated with the invasive condition may manifest after a prolonged period (⁓3.5 years) and may only emerge once the orbit or skull base has become affected [13]. The orbital penetration can lead to proptosis, which is the most common (88.2% frequency) manifestation in cases of CGIFRS, followed by sinonasal symptoms (e.g. nasal obstruction, nasal discharge, facial fulness) in 39.2% of cases [2]. Furthermore, damage to the cribriform plate could result in persistent headaches, seizures, or focal neurological symptoms with other associated symptoms (e.g. dizziness, vomiting, fatigue, and fever) [2, 14].

The early diagnosis of CGIFRS is imperative due to the potentially serious complications of the disease; a CT scan is recommended as the initial imaging modality with various possible findings (e.g. mucosal thickening and bony erosions). Additionally, the MRI is used to evaluate dural involvement and the intradural extension of the disease [15]. Nevertheless, it is challenging to differentiate CGIFRS from malignancy or other types of invasive fungal rhinosinusitis. Therefore, a definitive diagnosis of CGIFRS can only be achieved by distinctive histopathology (i.e. non-caseating granulomas with giant cells, vasculitis, and perivascular fibrosis) [15].

**Figure 5:**
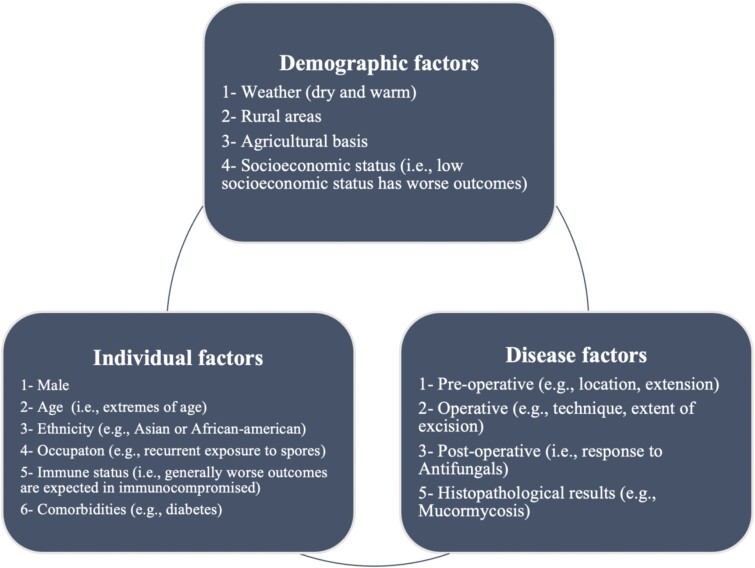
Prognostic considerations in CGIFRS [5, 10, 16].

The mortality rates for CGIFRS range from 5.9% to 22.2%, and the fatal consequences can occur between 3 and 17 months postoperatively [16]. In our study, the relapses occurred 3 weeks and 3 months postoperatively in the first and second patients, respectively. Several factors may increase the mortality rate for CGIFRS, including delayed diagnosis and treatment, the immunocompromised state of the patient, and the extent of the disease, which could determine the prognosis (Fig. 5).

Furthermore, Siddiqui *et al.* classified the presentation of invasive fungal rhinosinusitis into three patterns: intracerebral aspergillosis has the worst prognosis, intracranial extradural aspergillosis has an intermediate prognosis, and orbital and cranial base aspergillosis have the most favorable prognosis [17]. In a previous study that involved patients with acute invasive fungal rhinosinusitis, the researchers found that the patients with intracranial involvement had a poor prognosis [18]. Similarly, the intracranial invasion presented in our cases eventually led to death. Another factor that affects the prognosis in these patients is the treatment plan. Although surgical debridement with antifungal therapy is the primary strategy to treat CGIFRS, no definitive treatment protocol has been established, as discussions regarding the proper surgical approach and the best type, duration, and route of antifungal therapy are ongoing [4, 9, 13].

In some cases, an improper treatment plan due to inadequate surgical debridement or an ineffective response to antifungal therapy may result in mortality. Rupa *et al.* recently published a treatment protocol for CGIFS based on a staging system that utilizes the clinical presentation and radiological findings to assess the extent of the disease, as shown in Table 1 [9]. A study found that CGIFRS recurs in 8.7% of individuals, which may contribute to the increased mortality in these patients. However, no research has investigated the association between recurrence and mortality rates [2]. Notably, the differences in mortality rates across the different studies could be attributed to the ambiguity surrounding the cause of death, as fatalities may also result from underlying medical conditions rather than solely from CGIFRS.

**Table 1:** Rupa *et al.* treatment protocol for CGIFS [9]

Stage	Involvement	Suggested Intervention
1	The disease is localized to the nose and paranasal sinuses.	Endoscopic excisional surgery **+** Antifungal therapies^a^
2	The disease extends to the orbit, palate, or oral cavity.	Endoscopic and/or open excisional surgery **+** Antifungal therapies^a^
3	The disease further extends into the brain, pterygopalatine fossa, cavernous sinus, cheek, or periorbital area.	Combined endoscopic and open approach, which includes craniofacial resection of the involved anterior skull base **+** Antifungal therapies^b^

^a^Itraconazole, posaconazole, Amphotericin B, caspofungin, or Voriconazole.

^b^Voriconazole is preferred.

## Conclusion

CGIFRS is a rare, gradually progressive disease with potentially fatal consequences. A high level of clinical suspicion, radiological imaging, and tissue biopsy are crucial for prompt diagnosis and treatment. The extent of the disease and the management protocol are among the factors that may increase mortality in CGIFRS. In this study, we reported two cases of CGIFRS in which the patients received intensive medical care but ultimately succumbed to the condition. This study, therefore, underscores the severity of CGIFRS as a potentially fatal disease.
